# Progressive multifocal leukoencephalopathy and immune reconstitution inflammatory syndrome in seven patients with sarcoidosis: a critical discussion of treatment and prognosis

**DOI:** 10.1177/17562864211035543

**Published:** 2021-07-31

**Authors:** Maike F. Dohrn, Gisa Ellrichmann, Rastislav Pjontek, Carsten Lukas, Jens Panse, Ralf Gold, Jörg B. Schulz, Burkhard Gess, Simone C. Tauber

**Affiliations:** Department of Neurology, Medical Faculty of the RWTH Aachen University, Pauwelsstr. 30, Aachen, 52074, Germany; Department of Neurology, St. Josef-Hospital, Ruhr-University Bochum, Bochum, Germany; Department of Diagnostic and Interventional Neuroradiology, Medical Faculty of the RWTH Aachen University, Aachen, Germany; Department of Neurosurgery, Medical Faculty of the RWTH Aachen University, Aachen, Germany; Department of Radiology, St. Josef-Hospital, Ruhr-University Bochum, Bochum, Germany; Department of Oncology, Hematology and Stem Cell Transplantation, Medical Faculty of the RWTH Aachen University, Aachen, Germany; Department of Neurology, St. Josef-Hospital, Ruhr-University Bochum, Bochum, Germany; Department of Neurology, Medical Faculty of the RWTH Aachen University, Aachen, Germany; Jülich Aachen Research Alliance (JARA) – JARA-BRAIN Institute Molecular Neuroscience and Neuroimaging, FZ Jülich and RWTH University; Department of Neurology, Medical Faculty of the RWTH Aachen University, Aachen, Germany; Department of Neurology, Medical Faculty of the RWTH Aachen University, Aachen, Germany

**Keywords:** cidofovir, John Cunningham (JC) virus, lymphopenia, mefloquine, mirtazapine

## Abstract

Progressive multifocal leukoencephalopathy (PML) is a subacute brain infection by the opportunistic John Cunningham (JC) virus. Herein, we describe seven patients with PML, lymphopenia, and sarcoidosis, in three of whom PML was the first manifestation of sarcoidosis. At onset, the clinical picture comprised rapidly progressive spastic hemi- or limb pareses as well as disturbances of vision, speech, and orientation. Cerebral magnetic resonance imaging showed T2-hyperintense, confluent, mainly supratentorial lesions. Four patients developed punctate contrast enhancement as a radiological sign of an immune reconstitution inflammatory syndrome (IRIS), three of them having a fatal course. In the cerebrospinal fluid, the initial JC virus load (8–25,787 copies/ml) did not correlate with interindividual severity; however, virus load corresponded to clinical dynamics. Brain biopsies (*n* = 2), performed 2 months after symptom onset, showed spotted demyelination and microglial activation. All patients had lymphopenia in the range of 270–1150/µl.

To control JC virus, three patients received a combination of mirtazapine and mefloquine, another two patients additionally took cidofovir. One patient was treated with cidofovir only, and one patient had a combined regimen with mirtazapine, mefloquine, cidofovir, intravenous interleukin 2, and JC capsid vaccination. To treat sarcoidosis, the four previously untreated patients received prednisolone. Three patients had taken immunosuppressants prior to PML onset, which were subsequently stopped as a potential accelerator of opportunistic infections. After 6–54 months of follow up, three patients reached an incomplete recovery, one patient progressed, but survived so far, and two patients died. One further patient was additionally diagnosed with lung cancer, which he died from after 24 months. We conclude that the combination of PML and sarcoidosis is a diagnostic and therapeutic challenge. PML can occur as the first sign of sarcoidosis without preceding immunosuppressive treatment. The development of IRIS might be an indicator of poor outcome.

## Introduction

Progressive multifocal leukoencephalopathy (PML) is a diffuse infectious disease of the central nervous system (CNS) with a disabling and potentially lethal course.^1,2^ The opportunistic John Cunningham (JC) virus lingers in kidneys or lymphatic tissues in about 65–90% of the healthy population.^3^ First described in 1958 in a patient, after whom (John Cunningham) the virus was named,^1^ PML gained further recognition in the early 1980s, when it occurred more frequently in the context of the acquired immunodeficiency syndrome (AIDS) pandemic. PML can also occur in other conditions of immunosuppression such as malignancies or immuno-suppressant/-modulatory treatment of autoimmune disorders such as multiple sclerosis. Sarcoidosis – a granulomatous disease of not yet fully understood aetiology – has already been described to first present with symptoms of PML in very rare cases.^4,5^ A potential pathophysiological link between these two diseases appears to be a marked T-cell suppression related to sarcoidosis,^6,7^ the origin of which, however, remains unclear to date. Besides the exhaustion of regulatory T cells (Tregs) and consequent failure to suppress the increased Th1 and Th17 response, impaired antimicrobial response is an alternative way to explain immunosuppression in sarcoidosis.^8^

As both a pre-existing medical immunosuppression and sarcoidosis itself have the potential to cause PML, it is not clear to date how to balance the depth of iatrogenic *versus* disease-mediated immunosuppression. Furthermore, there are no recognized antivirals that have provided any benefit in the treatment of PML in randomized controlled trials. Mirtazapine,^9,10^ mefloquine,^11,12^ cidofovir,^13,14^ interleukin 2, and JC capsid vaccination all constitute experimental concepts,^15,16^ which have been tested only in small and heterogeneous patient collectives, most of which with a human immunodeficiency virus (HIV) infection as the underlying cause of immunosuppression and with varying results. Systemic IL7 treatment seemed to improve PML in several reports^17^; however, a subsequent flare of sarcoidosis was observed in a patient with the sarcoidosis/PML combination.^18^ Novel single-case approaches, including JC virus-specific T cells or immune checkpoint inhibitors, aim at re-establishing immunity to the virus rather than being antivirals in the traditional sense.^19,20^

An overwhelming immune response directed against an opportunistic agent such as JC virus can occur when the underlying immunosuppressant condition caused by HIV, tuberculosis, or leprosy^21,22^ is treated successfully, or administration of natalizumab – an α-integrin antibody preventing leukocyte migration across the blood-brain-barrier – is stopped.^23^ The so-called immune reconstitution inflammatory syndrome (IRIS) presents with a worsening of neurologic deficits accompanied by inflammatory neuroimaging changes such as lesion growth or dissemination and contrast enhancement.^24–26^

In the present study, we describe seven individuals with PML and sarcoidosis. In three of them, the presenting PML symptoms were the very first clinical manifestation, two were diagnosed with sarcoidosis but treatment-naive, while further two patients had already received immunosuppressant drugs due to sarcoidosis prior to developing any neurological symptoms. In three patients with a particularly poor outcome, we discuss the prognostic role of IRIS based on its clinical and radiological diagnosis. We depict the diagnostic and therapeutic challenge in such a rare combination of conditions and critically discuss the individually chosen treatment regimens within the frame of the current literature.

## Patients and methods

### Patient selection

To elaborate typical patterns or differences in the disease course, we retrospectively evaluated all available clinical, paraclinical, and treatment data belonging to seven individuals that were diagnosed with the rare combination of PML and sarcoidosis at two German centers. Treatment concepts including the choice of medication and the individual dosages were based neither on randomized, controlled study protocols nor on current guidelines, but on the local experts’ choice. The study was conducted according to the Declaration of Helsinki and has been approved by the local ethical committee of the RWTH Aachen University (EK 073-21).

### Examinations performed

In all patients, we assessed a detailed patient history including both symptoms of PML, such as visual field defects, subacute paresis or sensory loss, impairments of speech or swallowing function, disorientation or cognitive impairment of other quality, and signs, symptoms, or previous diagnostics of sarcoidosis (Table 1). Our clinical examination contained a complete neurological status, focussing especially on a potentially decreased strength or increased tone of muscle groups, brisk or asymmetrical tendon reflexes, sensory deficits, quadrant or hemianopia, aphasia, dysarthria and/or dysphagia. The following laboratory tests were assessed if available: overall leucocytes, absolute and relative lymphocytes, total T-cell count, CD4^+^ T-cells, CD8^+^ T-cells, Epstein-Barr virus (EBV) serology, quantiferon test, angiotensin converting enzyme (ACE), soluble interleukin 2 receptor, aspartate aminotransferase (ASAT), alanine aminotransferase (ALAT), and gamma-glutamyl transpeptidase (gamma-GT). In the cerebrospinal fluid (CSF), cell count, protein content, glucose level and lactate were measured. In all patients except for one (patient 3), the oligoclonal bands were assessed as well. At the baseline visit, JC virus was measured in CSF of all patients. Some patients had a follow-up spinal tap after 3 months (Table 1), and some additionally after 6 months. Both antibody-specific indices and JC virus polymerase chain reactions (PCR) were performed at the Institute of Virology of the Heinrich Heine University in Düsseldorf, Germany. Brain biopsies had been obtained and locally evaluated in two patients. The respective reports are summarized in the results section.

**Table 1.** Clinical findings, laboratory and MRI data.^a^.

Patient ID/sex	1/m	2/m	3/m	4/f	5/f	6/m	7/m
Decade at onset	5th	6th	6th	5th	7th	6th	4th
Previous history of sarcoidosis	No	Yes: hepatic	No	Yes: pulmonary, lymphadenopathy, Bell’s palsy, axonal sensorimotor polyneuropathy	Yes: pulmonary, hepatic, (supposedly) renal	Yes: pulmonary	No
Interval of PML-associated symptoms and diagnosis	~ 2 months	~ 2 months	~ 1 month	~ 1 month	~ 2 months	~ 1 month	~ 6 weeks
Interval of diagnosis to death (if applicable)	Alive	24 months	3 months	~ 4 months	Alive	Alive	Alive
Clinical information							
Motor examination	Brachiofacially accentuated right-sided hemiparesis	Left-sided hemiplegia	Right-sided hemiplegia, initially brachiofacially accentuated	Moderate tetraparesis accentuated on the right side	Initially brachially accentuated left-sided hemiparesis, developed hemiplegia and right-sided high-grade paresis in the course	Alien limb right arm	Right-sided hemiparesis
Deep tendon reflexes	Increased on the right	Increased on the left	Increased on the right	Increased, right > left	Increased on the left	Normal	Increased on the right
Pyramidal signs	None	Left positive Babinski’s sign	Right positive Babinski’s sign	Positive Babinski’s sign on both sides	None	None	None
Sensory deficit	According to paresis	None	Not assessable due to clinical condition	None	None	None	According to paresis
Visual field	Hemianopia to the right (initial symptom)	Hemianopia to the left (initial symptom)	Not assessable due to clinical condition	Normal	Cortical blindness	Normal	Normal
Coordination	Pronounced ataxia in the right arm	Bradydysdiadochokinesia	Not assessable due to clinical condition	Pronounced ataxia in the right arm, bradydysdiadochokinesia	Dysmetria in the right arm	Impaired fine motor skills	Not assessable due to paresis on the right
Disorientation	Fluctuating	Developed in the course of disease	Severe	Developed in the course of disease	Moderate cognitive deficits developed in the course of disease	Moderate in the course of disease	None
Aphasia	Mild expressive aphasia	None	Severe global aphasia	Severe global aphasia	None	None	Moderate primarily expressive aphasia
Dysarthria	None	Developed in the course of disease	Anarthria	Developed in the course of disease	Developed in the course of disease	Developed in the course of disease	Developed in the course of disease
Dysphagia	None	Developed in the course of disease	Severe	Developed in the course of disease	Developed in the course of disease	Developed in the course of disease	None
Severe aspiration	None	Possible	Severe	Severe	Possible	None	None
Lung function							
Vital capacity [5.04 l]	4.01	n.d.	2.18	n.d.	n.d.	3.17	n.d.
MRI scan							
Initial scan: interval from onset	2 weeks	4 weeks	4 weeks	5 days	7 weeks	4 weeks	8 weeks
Initial scan: findings	T2-hyperintense, confluent, subcortical lesion in the left occipital lobe, no Gd enhancement	T2-hyperintense, confluent, subcortical lesion in the right parietooccipital region, no Gd enhancement	T2-hyperintense, confluent, subcortical lesions in the left frontal and both parietal lobes, no Gd enhancement	10 new supra- and infratentorial T2-hyperintense lesions in both parietal lobes, temporo-occipital left lobe, and frontal right lobe, cortical and subcortical, four of them confluent, affection of subcortical U-fibres	T2-hyperintense confluent cortical and subcortical lesion in the right parietooccipital and temporooccipital region	T2-hyperintense cortical lesion in the left parietal region with additional T2 shine-through effect, affection of U-fibres in the left precentral region	T2-hyperintense confluent cortical and subcortical lesions in the left parietal, frontal, and parietooccipital region with surrounding oedema
Most recent scan: interval from treatment initiation	54 months	2 months	2 months	1 week	16 months	51 months	8 months
Most recent scan: findings	Subcortical sclerosis and asymmetric atrophy in the left occipital lobe, e vacuo extension of the left lateral ventricle	Progressive, bilateral, confluent T2-hyperintense lesions including basal ganglia, punctate Gd enhancement in the left parietal region	Progressive, bilateral, confluent T2-hyperintense lesions involving both hemispheres, diffuse punctate Gd enhancement in the left hemisphere	Progressive, bilateral confluent T2-hyperintense lesions involving both hemispheres, affection of U-fibres, Gd^+^, progression of cerebellar lesions and lesions in medulla oblongata	Progressive, bilateral and right dominated, confluent T2-hyperintense lesions including right external capsule, thalamus, and pyramidal track, affection of U-fibres, atrophy of the right hemisphere, progressive e-vacuo atrophy of the right lateral ventricle	Confluent, cortical T2-hyperintense lesions in the left hemisphere including thalamus, pons, left pedunculus cerebelli, and pontomesencephal; atrophy in the left hemisphere with e-vacuo extension of the left lateral ventricle	Enlargement of the multifocal, confluent left frontal and parietal lobe lesions
IRIS	No	Yes	Yes	Yes	Yes	No	No
CSF (baseline)							
Oligoclonal bands	Negative	Negative	n.d.	Positive	Positive	Negative	Negative,
Protein [0.13–0.4 g/l]]	0.46	1.06	0.35	0.62	0.80	0.78	0.37
Cell count [**<**4/µl]	1	1	1	1	1	2	1
JC virus count/PCR [0 c/ml]	25787	51	2610	21	8	90	4980
ASI [**<**1.5]	n.d.	n.d.	n.d.	0.63	0.45	3.31	n.d.
CSF (after 3 months)							
Oligoclonal bands	Positive	n.d.	n.d.	Positive	Positive	Negative	Positive
Protein [0.13–0.4 g/l]]	0.58	n.d.	n.d.	0.70	0.70	0.55	0.42
Cell count [**<**4/µl]	4	n.d.	n.d.	1	1	1	1
JC virus count/PCR [0 c/ml]	555	n.d.	n.d.	n.d.	0	0	220
ASI [**<**1.5]	13.9	n.d.	n.d.	n.d.	0.45	n.d.	n.d.
Further laboratory findings (baseline)							
Overall leucocytes [4200–9100/µl]	5200	3400	8200	6500	4300	5,100	6,800
Lymphocytes absolute [4200–9100/µl]	530	1110	990	1150	630	270	1,000
Lymphocytes relative [22–53%]	10.2	32.5	12.1	16.2	14.4	5.3	16.1
Total T-cell count [1100–1700/µl]	139	1014	983	–	476	83	450
CD4^+^ T-cell count [645–1289/µl]	106	464	404	–	382	70.9	132
CD8^+^ T-cell count [263–739/µl]	29	331	282	–	95	42.6	271
EBV (serology)	n.d.	n.d.	Positive (IgG)	n.d.	Negative	Negative	Negative
Quantiferon test	Negative	n.d.	Negative	n.d.	n.d.	n.d.	Negative
ACE [8.28 mU/ml]	24.3	n.d.	n.d.	19.4	72.5	21.5	13.7
sIL2-receptor [223–710 U/ml]]	664	55.6	820	429	3891	458	824
ASAT [<50U/l]	67	33	45	17	42	24	33
ALAT [<50U/l]	102	24	42	17	52	30	30
γ-GT [<60U/l]	215	245	133	21	244	34	25
Therapeutic regimes							
Therapy of sarcoidosis before PML	None	None	None	Azathioprine for 2 months, mycophenolate mofetil, infliximab for 3 months, fumaric acid for 3 months	None	Azathioprine 150 mg per day for 2 years	None
Therapy of sarcoidosis after diagnosis of PML	Prednisolone 50 mg 1-0-0 for four weeks, then monthly reduction of 10 mg, containing dosage: 5 mg 1-0-0	Prednisolone 5 mg 1-0-0 for 5 months, stop due to PML progression with immunosuppression	Prednisolone 500 mg 1-0-0 for 3 days, then prednisolone 100 mg 1-0-0 for 7 days, then prednisolone 50 mg 1-0-0 with originally planned monthly reduction of 10 mg, stop due to PML progression with immunosuppression	None	Initially none, Prednisolone 5 mg 1-0-0, 50 mg 1-0-0, slow reduction; containing dosage: 5 mg 1-0-0	None	Prednisolone 40 mg 1-0-0
Medication due to PML	Mirtazapine 45 mg 0-0-1, mefloquine 250 mg 1/week	Mirtazapine 60 mg 0-0-1, mefloquine 250 mg 1/week	Mirtazapine 30 mg 0-0-1, mefloquine 750 mg 1-0-0, then mefloquine 250 mg 1/week	Cidofovir 5 mg/kg bw (250 mg) twice in an interval of 7 days	Mirtazapine 60 mg 0-0-1, mefloquine 250 mg 1/week, cidofovir 5 mg/kg bw for 3 months	Mirtazapine 60 mg 0-0-1, mefloquine 250 mg 1/week, cidofovir 5 mg/kg bw, 13 times, vaccination JCV, IL 2 once 500.000 U/m^2^ BSA, then for 79 days 1.000.000 U/m^2^ BSA	Mirtazapine 60 mg 0-0-1 for 5 months, mefloquine 250 mg 1/week (total 4 times), cidofovir 5 mg/kg bw, 15 times for 5 months
*Pneumocystis jirovecii* prophylaxis	Cotrimoxazole	–	Cotrimoxazole	–	–	–	–
Outcome	Incomplete recovery	Disease progression, death	Disease progression, death	Disease progression, death	Incomplete recovery	Severe disease progression	Incomplete recovery
Residual symptoms	Latent brachial paresis, mild right-sided spasticity with brisk tendon reflexes, ataxia and intentional tremor in the right arm, hemianopia	–	–	–	Tetraparesis with left-sided hemiplegia and mild right-sided paresis, hemihypesthesia, brisk tendon reflexes, improvement of dysphagia	Tetraparesis with high grade brachiofacially accentuated right-sided and mild left-sided hemiparesis paresis, positive Babinski’s sign	Mild right-sided spastic hemiparesis, fully ambulatory, normal speech

a If required, the reference values are given in square brackets with the corresponding units. Medications are listed by the drug names and not by the trade names. Based on the availability of information, dosages are not always specified in detail.

γ-GT, gamma-glutamyltransferase; ALAT, alanine aminotransferase; ASAT, aspartate aminotransferase; ASI, antibody-specific index; BSA, body surface area; bw, body weight; c/ml, copies per millilitre; CSF, cerebrospinal fluid; EBV, Epstein-Barr virus; Gd, gadolinium; IL2, interleukin 2; IRIS, immune reconstitution inflammatory syndrome; JCV, JC virus; MRI, magnetic resonance imaging; n.d., not determined; PCR, polymerase chain reaction; PML, progressive multifocal leukencephalopathy; sIL-2, soluble interleukine-2 receptor; U, unit(s).

All patients received the first cranial MRI within at least 2 months after the onset of symptoms, mostly at a radiological office or local hospital. For each patient, three to ten follow-up MRIs were performed. The frequency of the MRI controls depended on the clinical evolution ranging from 4 days (during hospitalisation) to 9 months (as follow up in setting of outpatient clinic). All MRIs included in this work were reviewed critically by the same experienced neuroradiologist. The standard protocol included axial T2-weighted turbo spin echo (TSE), T2-weighted fluid attenuated inversion recovery (FLAIR), native and contrast enhanced T1-weighted spin echo, T2-weighted fast field echo/T2*, diffusion weighted imaging with apparent diffusion coefficient (ADC) maps, coronal contrast enhanced T1-weighted spin echo, and sagittal T2-weighted TSE sequence with slice thickness from 3 mm to 5 mm using a 1.5T or 3T scanner from different manufacturers (Philips, Eindhoven, the Netherlands; Siemens, Erlangen, Germany). The protocols from other hospitals or radiology offices included mostly axial T2-TSE, axial/coronal or sagittal T2-FLAIR, diffusion weighted imaging (DWI)/ADC, axial and coronal or sagittal T1-SE with/without contrast enhancement. Additionally, 3D-T1-FFE, axial proton density (PD) weighted sequence or time-of-flight MR-angiography were performed. PML lesions were defined according to the literature.^25–27^ IRIS was suspected in case of lesion growth or dissemination, T1 conversion from hypo- to hyperintensity, and, most of all, contrast enhancement.^24–26^

## Results

Two out of seven patients were female. Ranging between 38 and 62 years, the mean age at PML onset was 51.4 years. The mean duration from the very first symptoms to the diagnosis of PML was 1.5 months. At the end of follow up, four patients were still alive. Two patients died due to PML. One patient (patient 2) died from lung cancer 2 years after the diagnosis of PML. When being diagnosed with PML, no underlying malignancy was known, and a positron emission tomography–computed tomography (PET-CT) revealed only bilateral roundish compactions in the apical lower lobes, which were evaluated as unspecific infiltrations possibly related to sarcoidosis at that time. Further competing immunosuppressant conditions other than sarcoidosis were excluded in all patients. In three cases (patients 1, 3, 7), the first symptoms of PML preceded the diagnosis of sarcoidosis. A more detailed summary of the patient histories can be found in the supplemental material.

### Clinical data

The most notable common feature in our patients’ clinical picture was the diversity of symptoms and its fluctuation. New symptoms were noticed with a subacute occurrence and sometimes disclosed only in a detailed clinical examination. Motor deficits were seen in all patients ranging from latent paresis to hemiplegia. Six out of seven patients had brisk tendon reflexes in the involved segment. Pyramidal signs were seen in three out of seven patients. Two out of seven patients had a corresponding sensory deficit. Coordination deficits were reported in all examined individuals ranging from impaired fine motor skills to pronounced ataxia. The visual field was impaired in three patients. Dysarthria became evident in six out of seven patients, dysphagia in five. Two patients contracted a nosocomial pneumonia putatively related to aspiration. Neuropsychological deficits such as disorientation were reported in six out of seven patients. Aphasia was observed in four patients, of whom the quality was predominantly expressive in two and global in another two patients. Its severity was classified as mild in one patient, as moderate in another, and severe in further two patients.

### MRI findings

**Figure 1.**
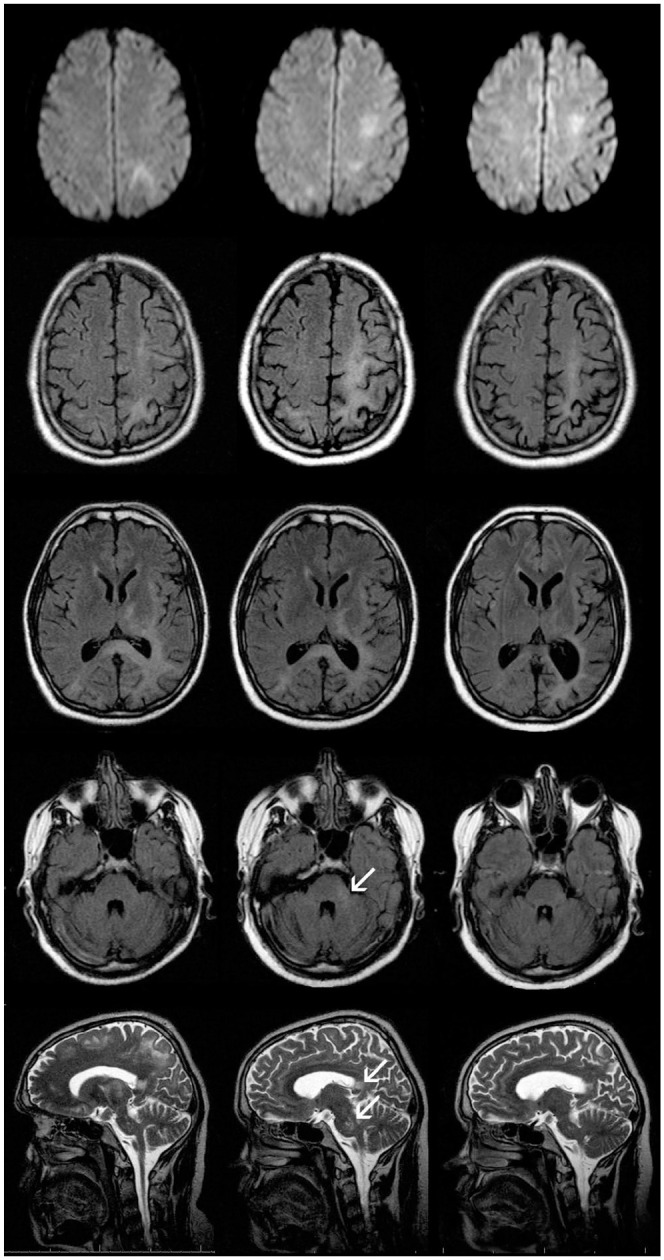
Axial DWI (upper row), T2-FLAIR (three middle rows) and sagittal T2-TSE (lower row) in patient 1. Left column shows images performed approximately 3 months after onset of the neurological symptoms (right sided homonymous hemianopsia and ataxia) and showing T2-hyperintense confluent subcortical lesions with left parieto-occipital focus but also involving left motoric and premotoric cortex, left parietal and occipital cortex, and subcortical white matter, posterior internal capsule, external capsule, and splenium of corpus callosum. In these areas, patchy diffusion restriction but no gadolinium enhancement is observed. The follow-up after 2.5 months (middle column) indicates progression of the subcortical PML lesions with left hemispheric accentuation (e.g. left cella media) but also new lesions in left pons and cerebellar pedunculus. Arrows indicate lesions in cerebellar pedunculus, splenium of corpus callosum, and pons. Approximately 6 months later (right column), decrease of the PML lesions with increasing atrophy and consecutive *e vacuo* enlargement of the inner CSF spaces can be noticed. Moreover, the infratentorial PML lesions are barely visible. CSF, cerebrospinal fluid; DWI, diffusion weighted imaging; PML, progressive multifocal leukoencephalopathy.

**Figure 2.**
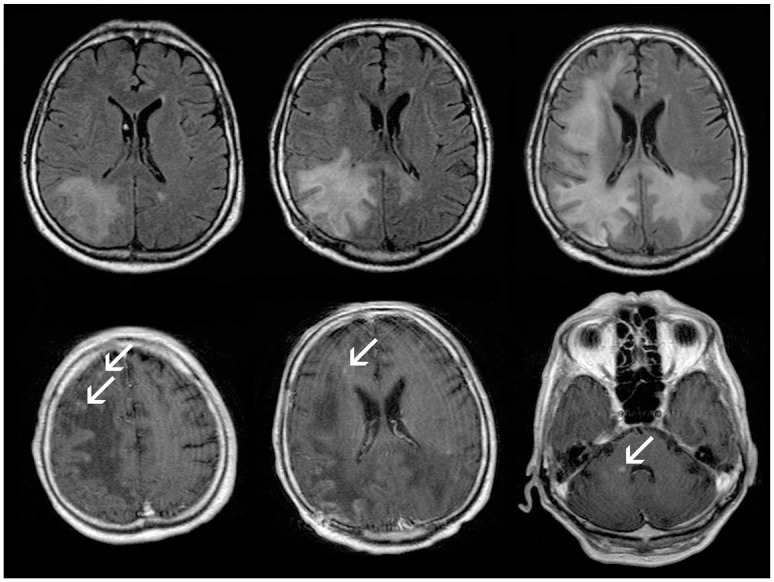
Follow-up (within 2 months) axial T2-FLAIR (upper row) and axial contrast enhanced T1-SE (lower row) in patient 2, extensive progression of posterior accentuated, mostly subcortical PML-lesions is observed (upper row). Approximately 3 months after onset of the neurological symptoms, punctuate contrast enhancement (arrows) appear at the borders of the PML lesions suggesting the development of IRIS, corresponding to further clinical deterioration (lower row). IRIS, immune reconstitution inflammatory syndrome; PML, progressive multifocal leukoencephalopathy.

PML lesions appeared T2-hyperintense, T1-hypointense, confluent, subcortical, and mainly supratentorial with a slight preference for frontal and parieto-occipital localization (Figures 1 and 2). U-fibres were affected in all cases, but not in all lesions. Three patients presented with a unilobar, two with multilobar (two to three lobes affected), and two with widespread (>3) PML manifestation on the initial MRI scan. All patients developed bilateral asymmetric widespread disease (within 1–13 months), which was attended by signal increase of splenium of corpus callosum on T2-weighted sequences. In all but one patient (patient 3), infratentorial lesions were observed, five of them involving brain stem and cerebellum, whereas one patient presented with an infratentorial lesion pattern similar to Wallerian degeneration. The thalamus, internal, or external capsule were affected in all patients. Acute PML lesions appeared hyperintense on DWI. Initially, no pathological gadolinium enhancement was detected. However, four patients developed contrast enhanced lesions in the course (Figure 2). IRIS was discussed in all of these four (patients 2, 3, 4, and 5) – three had a lethal course. Most of the lesions were punctate. In one case, the last pre-mortem MRI showed widespread punctate, rim-like, and circular contrast enhancement. A slight-to-moderate mass effect was seen in the last pre-mortem MRI in two (patient 3 and 4) patients with a lethal course. Long-term follow up of the survivors revealed asymmetric atrophies of the previously affected areas with an *e vacuo* phenomenon of the lateral ventricles (Figure 1).

### Histopathological findings

In two patients (patients 2, 5), we had access to the histopathological results of a brain biopsy, both obtained about 2 months after symptom onset. In both biopsies, the white matter showed spotted demyelination. In patient 2, the texture was roughly disturbed, but not necrotic, whereas the axonal network appeared mainly preserved in patient 5. Astrocytes were tightly stacked and diffusely distributed, with a swollen (gemistocytic) appearance and tenuous, multipolar extensions. Within the frame of an extensive microglial activation merging into loose macrophage niduses (CD68^+^), there was no evident lymphocytic infiltration (CD45/LCA) in patient 5, but small, CD8^+^ lymphocytes in the lesion periphery in patient 2. Macrophages showed partially enlarged, polymorphous nuclei with positive markers of proliferation and mitosis (Ki67, MiB-1, p53). In patient 2, the JC virus antigen SV40 was histologically confirmed. Patient 5 had 351,000,000 copies/µg of JC DNA in the brain specimen. Both biopsies did not indicate the development of IRIS at the time of sample obtainment.

### Laboratory data

Replication of JC virus was confirmed by PCR in CSF in all patients at baseline except for patient 6, in whom the PCR result was positive 3 months after onset only. The greatest virus load was observed in patient 1 with 25,787 copies/ml. Comparing all patients, the virus load did not appear to correlate with disease severity; however, individually, the clinical restitution corresponded to the reduction of intrathecal JC copies. Patient 1, 5, and 7, those with the best clinical outcomes, showed a marked reduction of JC replicates (patient 1: 25,787 copies/ml at baseline, 555 copies/ml after 3 months and 18 copies/ml after 6 months; patient 5: 8 copies/ml at baseline and 0 copies/ml after 3 and 6 months; patient 7: 4,980 copies/ml at baseline, 220 copies/ml after 3 months and 18 copies/ml after 6 months). Paralleling his virus load (90 copies/ml at baseline, 0 copies/ml after 3 months and 89 copies/ml after 6 months), patient 6 stagnated or showed secondary progression in his disease course. The oligoclonal bands were positive in two out of six examined patients right at the baseline visit. In patient 7, they turned positive after 3 months, remaining positive in the 6 months follow-up examination as well. Patient 5 had positive oligoclonal bands after 24 months. Patient 1 had no intrathecal IgG synthesis at baseline. He had positive oligoclonal bands after 3 months and a mild disruption of the blood–brain–barrier at 6 months follow-up. A protein elevation in CSF was seen in five out of seven patients at the baseline visit, in all four patients after 3 months and in two out of three examinations after 6 months. The overall cell count in the CSF was normal in all of the examinations.

Analysing the peripheral blood, an absolute lymphopenia was observed in all patients (Table 1). In contrast to all other individuals, patient 2 had an overall reduction of leukocytes resulting in a normal percentage, but absolute reduction of lymphocytes. This could link to the deviating clinical course as discussed in the context of competing reasons other than sarcoidosis responsible for immunosuppression. The absolute T-cell count was reduced in five out of six patients. CD4^+^ T-helper-cells were reduced in all patients, while CD8^+^ T-killer-cells were diminished in three out of six examinations. Table 2 exemplarily depicts the immunophenotyping results obtained from patient 1. All types of lymphocytes were low at all points of measurement; however, CD4^+^ and CD8^+^ T-cells were particularly reduced with a moderately elevated CD4^+^/CD8^+^ ratio. After treatment initiation with prednisolone, the absolute lymphocyte and T-cell count increased with a declining CD4^+^/CD8^+^ ratio, while B-cell counts slightly diminished. Two months after reducing the prednisolone dosage, total lymphocytes, B- and T-cells were even lower than before treatment. This appeared to stabilize within the following 5 months, so that, at the last control, 4 years after the first diagnosis of PML and sarcoidosis, all values had slightly improved, but not completely recovered (Table 2).

**Table 2.** Immunophenotyping in patient 1: At the time of first presentation, the T and NK cells were particularly low, and the IL2 receptor expression was above the reference level. After 8 months of prednisone treatment, the T cell levels had slightly improved, but the B cells subsequently declined significantly. At 2 months after dose reduction, the T cells had minorly decreased again, whereas the Il2 receptor expression had increased.

	Initial	+8 months^a^	+14 months^b^	+16 months	+2 years	+4 years	Reference level
Total leucocytes [/µl]	6000.0	7500.0	7500.0	7300.0	5200.0	5600.0	4600–7100/µl
Lymphocytes abs [/µl]	**480.0** (↓)	**510.0** (↓)	**442.0** (↓)	**533.0** (↓)	**598.0** (↓)	**640.0** (↓)	1600–2400/µl
Lymphocytes rel [%]	**8.0** (↓)	**6.8.** (↓)	**5.9** (↓)	**7.3** (↓)	**11.5** (↓)	**11.5%** (↓)	28–39%
B lymph CD19+ abs [/µl]	**187.0** (↓)	**51.0** (↓)	**58.0** (↓)	**91.0** (↓)	**120.0** (↓)	209	200–400/µl
B lymph CD19+ rel [%]	39.0	**10.0 (↓)**	13.0	**17.0 (↑)**	**20.0 (↑)**	**26.1 (↑)**	11–16%
T lymph CD3+ abs [/µl]	**139.0 (↓)**	**163.0 (↓)**	**111.0 (↓)**	**139.0 (↓)**	**221.0 (↓)**	**409.0 (↓)**	1100–1700/µl
T lymph CD3+ rel [%]	**29.0 (↓)**	**32.0 (↓)**	**25.0 (↓)**	**26.0 (↓)**	**37.0 (↓)**	**51.3 (↓)**	67–76%
TH CD3+ CD4+ abs [/µl]	**106.0 (↓)**	**112.0 (↓)**	**80.0 (↓)**	**107.0 (↓)**	**173.0 (↓)**	**331.0 (↓)**	700–1100/µl
Tsuppr CD3+CD8+ abs [/µl]	**29.0 (↓)**	**46.0 (↓)**	**35.0 (↓)**	**43.0 (↓)**	**54.0 (↓)**	**58.0 (↓)**	500–900/µl
Quot CD4/CD8	**3.7 (↑)**	**2.4 (↑)**	**2.3 (↑)**	**2.5 (↑)**	**3.2 (↑)**	**5.7 (↑)**	1.0–1.5
Quot CD11b-/11b+ CD8	0.8	0.4	0.2	0.3	0.6	nd	<3
Ttox CD11b-CD8+ [/µl]	34.0	41.0	18.0	37.0	66.0	nd	
T suppr CD11b+CD8+ [/µl]	43.0	97.0	106.0	123.0	114.0	nd	
NK CD57 CD8 [/µl]	**48.0 (↓)**	**87.0 (↓)**	**97.0 (↓)**	**96.0 (↓)**	**66.0 (↓)**	nd	100–360/µl
NK C16 CD56 [/µl]	**144.0 (↓)**	275.0	212	298.0	269.0	nd	200–400/µl
HLA DR expr T CD3+ [%]	**36.7 (↑)**	**29.0 (↑)**	**35.7 (↑)**	**31.2 (↑)**	**21.6 (↑)**	nd	8–15%
IL 2R expr CD25 CD3 [%]	**55.2 (↑)**	**53.1 (↑)**	**60.0 (↑)**	**57.7 (↑)**	**51.4 (↑)**	nd	13–24%

a After 8 months of treatment with prednisone, mefloquine, and mirtazapine.

b At 2 months after dose reduction of prednisone.

Pathological values are bold. Arrows depict whether a value is above or below the reference level.

−, negative; +, positive; abs, absolute; expr, expression; HLA, human leucocyte antigen; IL2R, interleukin 2 receptor; lymph, lymphocytes; NK, natural killer; quot, quotient; rel, relative; TH, T helper cells; Tsuppr, T suppressor cells; Ttox, cytotoxic T cells.

As a typical feature of sarcoidosis, angiotensin converting enzyme was elevated in all patients examined (*n* = 5). The soluble interleukin (IL) 2 receptor, as a marker for T-cell activation, however, was elevated in only three out of seven patients, possibly reflecting an insufficient T-cell response. An elevation of liver enzymes, mostly gamma-GT, was seen in five out of seven patients at the baseline visit.

### Therapeutic regimens

In the described patient cohort, the therapeutic concept was based on three columns: JC virus inhibition, immunosuppressant adjustment, and *Pneumocystis jirovecii* prophylaxis.

#### JC virus inhibition

Except for patient 4, all patients received a combination therapy with mirtazapine and mefloquine. The mirtazapine dosage varied between 30 and 60 mg/day and was adapted to the patient’s individual side effects such as fatigue and vertigo, but also to liver and kidney function. Mefloquine was given once per week in a dosage of 450 mg. Additionally, patients 5, 6, and 7 received the antiviral drug cidofovir at a dosage of 5 mg/kg body weight (Table 1). As she refused other substances, patient 4 received a monotherapy with cidofovir without an additional mirtazapine or mefloquine treatment. Patient 6 additionally received a JC virus capsid vaccination and intravenous IL2 in a dosage of 500,000 units/m^2^ body surface area (BSA) on the first day and 1,000,000 units/m^2^ BSA for the following 79 days.

#### Immunosuppressant adjustment

Considering that sarcoidosis was the cause of T-cell-deficiency, a slight immunosuppression with prednisolone was started in all patients with no prior treatment of sarcoidosis (patients 1, 2, 3, 5, and 7). The dosage was chosen individually based on the interdisciplinary recommendations of involved pulmonologists, haematologists, and neurologists. Two patients had already undergone immunosuppression prior to PML diagnosis, one with azathioprine only (patient 6) and one with a subsequent combination of azathioprine, mycophenolate mofetil, infliximab, and fumaric acid (patient 4), so that this was assumed a competing cause of T-cell-suppression. In both cases, the immunosuppressant treatment was stopped immediately after diagnosing PML.

Additionally, two patients (patient 1 and 3) received a prophylaxis with cotrimoxazole against the opportunistic fungus *P. jirovecii*. In all cases, supportive care was provided by in- and out-patient physio-, occupational, and speech therapists, and specialized rehabilitative staff.

### Outcomes

Out of seven examined patients, three reached an incomplete recovery. Remaining symptoms were a mild-to-moderate spastic hemi- or tetraparesis and defects of the visual field, whereas dysarthria, dysphagia, and neuropsychological deficits recovered (almost) completely. All these three patients had not received any immunosuppressant treatment before the diagnosis of PML. Throughout all follow-up examinations, sarcoidosis remained the only notable reason for T-cell-depletion.

With an already diagnosed and azathioprine-treated sarcoidosis, patient 6 developed a severe disease progression in spite of multimodal medical therapy with mirtazapine, mefloquine, cidofovir, intravenous IL2, and JC virus vaccination. He now has high-grade tetraparesis with a right-sided accentuation and severe aphasia. Azathioprine has been stopped, which has so far not led to a severe progression of sarcoidosis.

Within this examined group, patients 3 and 4 had the worst clinical condition already at first contact. Whereas patient 3 had so far been untreated and sarcoidosis unknown, patient 4 had already received a long-term immunosuppression with azathioprine, mycophenolate mofetil, infliximab, and fumaric acid. As each of these medications is a risk for opportunistic infections, immunosuppression was paused after diagnosis, and an antiviral treatment with mirtazapine, mefloquine, and cidofovir initiated. After 4 months of unstopped disease progression, however, both patients died as a consequence of PML. Pre-mortem MRIs showed punctate contrast enhancement as potential signs of IRIS.

Similarly, patient 2 had been diagnosed with sarcoidosis prior to PML manifestation. With a so far pure hepatic manifestation, the only immunosuppressant treatment was prednisolone. With mirtazapine and mefloquine, his clinical status worsened, but spontaneously stabilized even after stopping all types of medication in a palliative setting. What he died of, however, was not PML or an associated complication, but a newly diagnosed adenocarcinoma of the lungs.

## Discussion

Immunosuppression is the common cause of PML enabling the ubiquitously lingering JC virus to infect the CNS. It is, therefore, not surprising that PML is typically associated with conditions such as AIDS, chemotherapy, or long-term immunosuppression in autoimmune diseases such as multiple sclerosis.^28,29^

A coincidence of PML and sarcoidosis was first described by Christensen and Fog in 1955.^30,31^ Before naming an ‘association’ between the two diseases, PML appeared to be an incidental autopsy finding in the first six cases mentioned in the literature, whereas the related symptoms had been mistaken for CNS sarcoidosis.^30^ As an independent disease entity, PML was first described in 1958.^1^ To date, only a few reports are available, most of them including less patients than in this study.^4,5,32^ Comparing our patients with the largest cohort (*n* = 10), reported by Jamilloux and colleagues,^5^ there were notable similarities in age (51.4 *versus* 51.6 years) and sex distribution (29% *versus* 20% female). Sarcoidosis was purely mediastino-pulmonary in 60% of the cases, while additional organ manifestations were observed in 43% of our cohort. Prior to PML, 40% of the Jamilloux cohort and 71% of our patients were naive to immunosuppressants. PML was the first manifestation of sarcoidosis in 20% of the other and in 43% of our cohort. With 3.6 months after first neurological symptoms, the mean time to PML diagnosis was slightly longer than in our cohort (1.5 months), and neurosarcoidosis – a common misdiagnosis reported by Jamilloux and colleagues – did not lead to wrong or delayed treatment in our patients. Considering that the death rate attributed to PML is higher in the previously reported patients (60% *versus* 29%), some correlation can be observed between a later diagnosis and poor outcome. In our cohort, patient 3 had the most aggressive clinical course and the shortest life span after onset. This might have accelerated the diagnostic decision making, so that the aforementioned considerations particularly apply for patients with an initially slower progression and thereby unspecific clinical course. Manifestation patterns are, indeed, another parallel between the two cohorts, as they include motor, sensory, visual, and cognitive systems, affect both hemispheres, and do not respect vascular territories.

What is it, after all, that causes the liability to PML in patients with sarcoidosis? Since PML has been observed in untreated sarcoidosis patients, a primary relation seems likely. Disease activity of sarcoidosis, reflected by organ manifestations or biomarkers such as sIL2-receptor,^1,2^ seemed, however, not to correlate with PML onset and course: In two out of three patients with elevated sIL2-receptor, sarcoidosis was clinically inapparent and in another patient stably controlled without treatment when PML occurred. In our first patient, a previously healthy 44-year-old man, the inspection of all former blood counts revealed a marked leap in the peripheral lymphocytes prior to his first presentation (supplemental material). T-cell suppression in fact turned out to be a common feature in all our patients independent of prior immunosuppressive medication. In the literature, sarcoidosis, a granulomatous disease with major T-cell involvement,^33,34^ is known to cause decreased CD4^+^, CD8^+^, or CD19^+^ counts in up to 70% of patients,^35,36^ which seems to correlate with disease severity.^36^ CD4^+^ levels have, however, a broad range and, on average, lie above the expected cut-off in AIDS patients. In our cohort, they were all below 500/µl (range: 106–464/µl), whereas this initial count did not correlate with the PML course or overall outcome. Considering this, there might be other influential factors interlinking sarcoidosis and PML that go beyond T cell counts alone.^37^ For treatment considerations, it appears reasonable to monitor and control lymphopenia, e.g. by balancing treatment of sarcoidosis with its immunosuppressant effect, thus reducing the risk of JC virus spread through treatment itself.

### Prognostic value of MRI and CSF

The prognosis of PML depends mainly on the reversibility of immunosuppression. An overwhelming immune reconstitution can, however, manifest as a subacute encephalitis with neurologic deterioration corroborated by new MR-tomographic signs of inflammation. The phenomenon IRIS has previously been described in the context of opportunistic infections in AIDS patients and in other immunosuppressive constellations such as natalizumab treatment. Associated with sarcoidosis, in which PML is a very exceptional manifestation itself, IRIS has been described only very few times.^18,38^ In the present study, cranial MRIs revealed signs of IRIS in precisely those patients with an unfavourable outcome. The absolute JC virus count in the CSF did not correlate with the respective patient’s symptom severity, but its trend did coincide with the individual disease course. This is consistent with the literature.^39^ Our results therefore suggest that the development of IRIS with its clinical exacerbation is associated with high morbidity and mortality and not solely the presence of opportunistic JC virus is an important cause of death in patients with PML associated to sarcoidosis.

### Therapeutic struggling: a lack of evidence

The present study has an observational, retrospective design. All treatment regimens were conceptualized individually according to the local experts’ opinion. Comparing the two different contributing centres, it becomes evident that patients 4–7 received additional medication with cidofovir, whereas patients 1–3 were given only mirtazapine and mefloquine. In one especially severe case (patient 6), IL2 was given intravenously for two cycles of 3 months each on top of the medication noted above, and a vaccination with a JC virus capsid peptide was performed. In one, but not both centres, a *P. jirovecii* prophylaxis with cotrimoxazole was implemented. This is due to the fact that no guidelines are available, and all considerable treatment options base their evidence on a scattered handful of case descriptions.

If sarcoidosis is the only reason for immunosuppression, can immunoregulatory treatment even be considered a therapy or is it more of an additional burden? This and many other questions remain unanswered. In 2010, Crouser *et al.* showed that lymphocyte counts can increase in sarcoidosis patients treated with antibodies directed against tumor necrosis factor alpha,^40^ whereas infliximab treatment itself has been discussed as a risk factor for PML development.^41^ In our cohort, we decided to initiate a systemic corticosteroid treatment in all patients that were previously naive to immunosuppressants. This harbored the risk of additional opportunistic infections or PML worsening.^42^ In these patients, we observed PML improvement in three, stabilization in one, and deterioration and death in one additional patient. A clear relationship between corticosteroid response and any of these outcomes can, however, not be established, as additional JC virus-directed drugs were applied, and both the time point and clinical status at treatment onset varied.

Mirtazapine has been recommended in a case series of four HIV patients with PML, out of whom three showed an improvement in clinical examination and one patient even in the MRI scan.^9^ As the underlying mechanism, an inhibition of serotonin receptors has been discussed as being essential for CNS infection.^43^ In their retrospective study on a total of eight treated patients, Jamilloux and colleagues described a significant decrease of mortality risk in sarcoidosis patients with PML [hazard ratio (HR) = 0.12, confidence interval (CI) 95% 0.02–0.9, *p* < 0.013].^5^ Mefloquine was identified to have anti-JC-viral activity *in vitro* while being able to sufficiently penetrate into the CNS.^11^ With a putatively moderate response, however, it has been used in a few individuals only (e.g. Ellrichmann et al.^12^). In another case report, a combined therapy with both mirtazapine and mefloquine was hypothesized to induce a cognitive improvement in a patient with HIV and PML.^44^ In one patient with sarcoidosis-related PML, an additional treatment with cidofovir appeared to be beneficial.^14^ Notwithstanding, the broadest experience in PML treatment originates from AIDS patients, in which cidofovir was discussed to be effective already in the early 2000s.^45,46^ A pooled multicohort analysis with 370 patients, however, did not show a statistically relevant influence of cidofovir on PML mortality and residual disability.^13^ Being produced by activated T cells and influencing peripheral CD8 cell counts, the rationale for IL2 treatment was its potential to influence the anti-JCV cytotoxic T-cell response. This therapy regimen has been used successfully in PML in Hodgkin’s lymphoma and myelodysplastic syndrome.^15,47^ In 2016, Dubey *et al.* described IL2 treatment in a natalizumab-associated PML.^48^ Evidence, though, is still lacking particularly with respect to long-term responses.

Similar to the inconsistency of literature, the choice of treatment varied markedly in our patients, too. In order to deduce comparisons, it may appear interesting to juxtapose the cidofovir-treated and -untreated patients. Yet, of course, the number of patients is small. In fact, out of both cohorts, two individuals passed away in the course of PML. Another patient, who died from lung cancer 2 years after diagnosis, should perhaps not be taken into account in terms of treatment response as the concomitant malignancy has been a putative confounder. Two patients with and one patient without cidofovir additional to their medication with mirtazapine and mefloquine reached herein the best possible outcome of incomplete, but stable, recovery. As a common feature in all these three, it needs to be added that no immunosuppression had been applied before PML manifestation, and prednisolone took part of the therapeutic regimen. It is therefore difficult to estimate whether cidofovir contributed effectively to this favourable outcome. Our results rather suggest that a pre-existing immunosuppression and a poor patient status at first diagnosis are further relevant predictors of a dismal prognosis in sarcoidosis-associated PML.

Pembrolizumab is a monoclonal antibody targeting the programmed death receptor on lymphocytes, a so-called immune checkpoint, inhibition of which results in the stimulation of the specific immune response. Approved for the treatment of various cancer subtypes, pembrolizumab has first been tested in a case series of six PML patients with variable underlying immunodeficiencies in 2019.^20^ Five out of eight patients showed a substantial decline of the CSF JC virus load, which was accompanied by clinical and radiological improvement. The one included patient with PML and sarcoidosis [female, 62 years old, previous immunosuppressive treatment with prednisone, methotrexate and tumour necrosis factor alpha (TNFα)-inhibitors) did not show a significant increase in CD4 cells reactive against JC virus antigens, nor did she respond clinically. Considering the effect of immune checkpoint inhibitors on sarcoidosis, a recent World Health Organisation (WHO) database report showed that several, albeit pauci-symptomatic first manifestations had been reported in such association.^49^ Therefore, we have not treated any of our patients included in the present study with pembrolizumab so far.

We conclude that PML can occur primarily in sarcoidosis without any preliminary medical immunosuppression. Considering the therapeutic implications, the containment of sarcoidosis on the one hand and the avoidance of an additional iatrogenic T-cell-weakening on the other remains a challenge for physicians. Treatment options aiming at reducing viral replication or uptake such as mirtazapine, mefloquine, cidofovir, JC virus vaccination, and IL2 substitution all constitute conceivable regimens; however, evidence is lacking for each of them.

## Supplemental Material

sj-docx-1-tan-10.1177_17562864211035543 – Supplemental material for Progressive multifocal leukoencephalopathy and immune reconstitution inflammatory syndrome in seven patients with sarcoidosis: a critical discussion of treatment and prognosis Click here for additional data file. Supplemental material, sj-docx-1-tan-10.1177_17562864211035543 for Progressive multifocal leukoencephalopathy and immune reconstitution inflammatory syndrome in seven patients with sarcoidosis: a critical discussion of treatment and prognosis by Maike F. Dohrn, Gisa Ellrichmann, Rastislav Pjontek, Carsten Lukas, Jens Panse, Ralf Gold, Jörg B. Schulz, Burkhard Gess and Simone C. Tauber in Therapeutic Advances in Neurological Disorders
