# Supplementary material for: Progressive multifocal leukoencephalopathy and immune reconstitution inflammatory syndrome in seven patients with sarcoidosis: a critical discussion of treatment and prognosis
Source: Ther Adv Neurol Disord. 2021 Jul 31;14:17562864211035543. doi: 10.1177/17562864211035543 (PMC8326823; doi:10.1177/17562864211035543)
Supplement: sj-docx-1-tan-10.1177_17562864211035543 – Supplemental material for Progressive multifocal leukoencephalopathy and immune reconstitution inflammatory syndrome in seven patients with sarcoidosis: a critical discussion of treatment and prognosis [file sj-docx-1-tan-10.1177_17562864211035543.docx]

**Progressive multifocal leukoencephalopathy and immune reconstitution inflammatory syndrome in seven patients with sarcoidosis: a critical discussion of treatment and prognosis**

Maike F. Dohrn^1^, Gisa Ellrichmann^2^, Rastislav Pjontek^3.4^, Carsten Lukas^5^, Jens Panse^6^, Ralf Gold^2^, Jörg B. Schulz^1,7^, Burkhard Gess^1^, Simone C. Tauber^1^

^1^Department of Neurology, Medical Faculty of the RWTH Aachen University, Aachen, Germany

^2^Department of Neurology, St. Josef-Hospital, Ruhr-University Bochum, Bochum, Germany

^3^Department of Diagnostic and Interventional Neuroradiology, Medical Faculty of the RWTH Aachen University, Aachen, Germany

^4^Department of Neurosurgery, Medical Faculty of the RWTH Aachen University, Aachen, Germany

^5^Department of Radiology, St. Josef-Hospital, Ruhr-University Bochum, Bochum, Germany

^6^Department of Oncology, Hematology and Stem Cell Transplantation, Medical Faculty of the RWTH Aachen University, Aachen, Germany

^7^JARA - Translational Brain Medicine

**Supplementary material**

*Patient histories*

Out of complete soundness, patient 1, a 44-year-old male, incurred a car accident. Homonymous hemianopia of the right visual field was subsequently identified as a correlate of broad, FLAIR-hyperintense subcortical white matter lesion most pronounced in the left temporal and occipital lobe. To a smaller extent, comparable lesions were found to be present in the left premotor and motor cortex and in the right parietal lobe (Fig. 1). Within this area, spotted regions of diffusion restriction were seen as have been typically described in PML patients. In the cerebrospinal fluid, JC virus was highly positive with 25,787 copies/ml. A peripheral blood cell count revealed a marked T-cell deficiency (absolute count: 139/µl [1100-1700/µl], Table 1). We excluded an infection with HI-virus. No immunosuppressant therapy had ever been applied in the past. Previous hemogram reports had already shown a leaping difference in the lymphocyte count 13 years ago. Re-evaluating the patient’s history, he had suffered from slight dyspnoea for at least seven years. A CT-scan now revealed streaky, scarred compacting areas in both lungs as well as enlarged, calcified mediastinal lymphatic nodes. Histologically, epitheloid, non-caseating granulomas were identified with negative stain for Ziehl-Neelsen. Intraabdominal lympathic nodes turned out to be enlarged as well. Hepatic enzymes were slightly elevated. We immediately started an oral treatment with prednisolone in a dosage of 40 mg per day, which was subsequently reduced in steps of 10 mg per month with a maintained dosage of 5 mg daily. Additionally, mirtazapine and mefloquine were applied. Due to immunosuppression, metronidazole was given as pneumocystis prophylaxis. In the meantime, the patient's status had worsened as he developed a right sided spastic hemiparesis, neuropsychological deficits, and disorientation, which all improved distinctly by treatment. After four and a half years of follow-up, the paresis had regressed to a latent stage, neuropsychological abilities had improved, and hemianopia remained stable. MRI scans showed asymmetric atrophies of the previously affected areas (Figure 1), and concentrations of JC virus in CSF decreased to 18 copies/ml. T-cell count, however, only improved slightly (Table 2).

Patient 1 is an elusive example of PML as first manifestation of sarcoidosis. Comparably, no previous history of sarcoidosis was known in patient 3 and 7. In case of the latter, the therapeutic regimen differed from the one described above in so far as cidofovir was additionally given for six months (Table 1). An incomplete recovery was observed as well. In contrast to these two patients, patient 3 had already presented in a markedly worse condition (Table 1). With a diagnostic latency of one month only, a treatment with prednisolone, mirtazapine, and mefloquine was initiated. Following an aspiration, however, the patient had to be transferred to the intensive care unit followed by intubation and tracheotomy. In the follow-up MRI, the white matter lesions progressed, showing spotted contrast enhancement as a potential sign of immune reconstitution inflammatory syndrome (IRIS). We reduced the prednisolone dosage and tried to improve the T-cell function by high-dose Vitamin C application. Unfortunately, the JC virus count remained at 2610 copies/ml. Four months after disease manifestation, the patient passed away on a palliative care unit.

As a pre-existing condition, sarcoidosis had already been known in patients 2, 4, 5, and 6. An immunosuppressant treatment had only been applied in two of these patients: azathioprine only in patient 6, and consecutive intervals with azathioprine, mycophenolate mofetil, infliximab, and fumaric acid without any overlap in patient 4. Out of these pre-treated patients, the one with the longer immunosuppressant history (patient 4) died four months after the development of PML-symptoms. While patient 6 is still alive, he, however, showed a severe progression even under an antiviral therapy with mirtazapine, mefloquine, cidofovir, JCV vaccination, and repeated interleukin-2 treatment. Patient 5, despite the already known sarcoidosis, never had received immunosuppressant treatment prior to PML onset. After diagnosis, however, we began a prednisolone therapy and an antiviral regimen with mirtazapine, mefloquine, and cidofovir. Thereby, he reached an incomplete recovery.

Patient 2 constitutes an exception in this patient cohort: The 56-year-old man first presented with non-directional vertigo, quadrant anopia, and occipital headache. Seven months before, a hepatic sarcoidosis had been diagnosed and since then he had been treated with oral prednisolone. With typical MRI lesions (Figure 2) and 51 copies/ml assessed by JC virus PCR from CSF, a cerebral biopsy finally confirmed PML. The clinical condition continuously worsened under treatment with mirtazapine and mefloquine. Under consideration of the patient’s will, a palliative setting was chosen, and all medication stopped. Surprisingly however, the disease stabilized for about two years, and the patient was able to sit, eat, and speak without help. When he eventually developed new respiratory symptoms, an advanced-stage adenocarcinoma of the lung was diagnosed, which he subsequently died from within a few months. Whether an already underlying malignancy had been a competing or additional cause of immunosuppression when PML was first diagnosed, remains unsolved. A PET-CT showed bilateral roundish compactions in the apical lower lobes, which were evaluated as unspecific infiltrations possibly related to sarcoidosis at that time. The patient’s relatives did not consent an autopsy.
